# A network meta-analysis of different acupuncture therapy in the treatment of poststroke cognitive impairment and dementia

**DOI:** 10.1097/MD.0000000000040233

**Published:** 2024-10-25

**Authors:** Lei Huo, Manli Zhao, Zeying Wang, Lunzhong Zhang, Kaili Fu, Xuezheng Zhang

**Affiliations:** a Weifang Hospital of Traditional Chinese Medicine, Weifang, China.

**Keywords:** acupuncture, ADL, dementia, MMSE, MoCA, network meta-analysis, poststroke cognitive impairment

## Abstract

**Background:**

Poststroke cognitive impairment and dementia (PSCID) is a major cause of stroke-related morbidities and mortalities. Over the last few years, there has been growing evidence supporting the effectiveness of needle-related treatments in PSCID. Our goal was to rate the included therapies and assess the clinical effectiveness of various needle-related treatments in patients with PSCID.

**Methods:**

We searched PubMed, Web of Science, Cochrane Library, Embase, China National Knowledge Infrastructure (CNKI), China Science and Technology Journal Database (VIP), Chinese Biomedical Literature Service System (SinoMed), Wanfang, FDA.gov, and ClinicalTrials.gov. A mix of subject terms and free words was used to search the databases. The retrieval period was from the inception date of the database to February, 2023. We included SRs and MAs from acupuncture RCTs of patients with PSCID. The Cochrane Risk Assessment Scale was used to evaluate the risk of bias in the included studies. State 17.0 was used for network meta-analysis in accordance with the Bayesian framework.

**Results:**

There were 34 studies total of 2690 patients. The cumulative ranking curve (SUCRA) revealed that CT + CFT + EA was the most efficient intervention to improve (Mini-Mental State Examination, MMSE) efficiency, followed by CT + CFT + AP to improve (Montreal Cognitive Assessment, MoCA) efficiency, CT + CFT + ACU for improving (Activities of Daily Living scale, ADL) scores, and CT + CFT + EA to improve clinical efficiency.

**Conclusion:**

The results show that Different acupuncture methods can improve cognitive function and daily living ability in patients with PSCID. Network meta-analysis revealed that both CT + CFT + ACU and CT + CFT + EA appeared to be more beneficial for daily living activities, while CT + CFT + EA and CT + CFT + AP appeared to be more helpful for cognitive performance in patients with PSCID. Treatments including acupuncture are safer and have a reduced incidence of negative side effects.

## 1. Introduction

Two different levels of cognitive impairment are included in poststroke cognitive impairment and dementia (PSCID): poststroke cognitive impairment with no dementia (PSCIND) and poststroke dementia (PSD).^[1]^ Acute ischemic stroke (IS),^[2]^ intracerebral hemorrhage (ICH),^[3–5]^ and aneurysmal subarachnoid hemorrhage (SAH)^[6,7]^ are other conditions that can lead to PSCID.

Stroke is the second most common cause of disability and death worldwide is stroke.^[8]^ Along with motor function deterioration, progression is accompanied by a reduction in cognitive ability.^[9]^ According to previous studies, the prevalence of PSCI is 80.97%. PSCID is a significant cause of morbidity and death following stroke worldwide.^[10]^ Their impact on patients’ capacity to take care of themselves and contribute to society was significant.^[11,12]^ The reported causes of dementia include other possible neuropathological processes, including perivascular spacing, impaired glymphatic system, cerebral amyloid angiopathy, microglial activation, vasogenic edema.

To date, there has been no unequivocally efficacious treatment for PSCID. Some studies have demonstrated some beneficial benefits of PSCID when taken in AD. Although studies have demonstrated that these drugs can help patients execute cognitive tasks better, the increase in general cognitive function and everyday functioning remains unclear.^[13]^ Therefore, new therapeutic options must be investigated.

In recent years, it has been demonstrated that PSCID patients who get acupuncture, a traditional Chinese medicinal therapy, had improved cognitive function.^[1,14]^ The most common types of acupuncture therapies include acupuncture, electroacupuncture, fire acupuncture, and warm acupuncture.

There are currently no studies evaluating the effectiveness of various acupuncture therapies; the existing meta-analyses exclusively contrast acupuncture with drugs or rehabilitation training.^[15]^ The network meta-analysis (NMA) approach works well for contrasting and rating the different treatments. As a result, the objective of this study was to identify the most effective technique for treating PSCID using acupuncture by evaluating the efficacy and safety of several acupuncture therapies.

## 2. Materials and methods

### 2.1. Methods

This NMA report follows the Preferred Reporting Items for Systematic Reviews and Meta-Analyses (PRISMA) Statement and is registered in the International Prospective Register of Systematic Reviews (number CRD42023398732).

### 2.2. Search strategy

We selected relevant studies published between January 1, 1950, and February 7, 2023, by searching PubMed, Embase, Cochrane, Web of Knowledge, CNKI, SinoMed, VIP, Wanfang databases, FDA.gov, and ClinicalTrials.gov. There were no language limitations to this study. Both subject words and free words were employed, such as stroke, apoplexy, cognitive dysfunction, cognitive impairment, cognitive disorder, acupuncture, electro-acupuncture, auricular acupuncture, fire needle, and randomized. Search strategies are provided in Appendix 1, Supplemental Digital Content, http://links.lww.com/MD/N786.

### 2.3. Inclusion criteria

We included SRs and MAs from acupuncture RCTs of patients with PSCID. Sex, age, and disease progression were not factors in PSCID diagnosis. Traditional acupuncture, fire needle therapy, electroacupuncture, head penetration needling, or acupuncture in combination with other therapies were all included in the treatment group. Other treatments beyond acupuncture were used in the control group, including sham acupuncture, placebos, conventional therapy, Western medicine, and cognitive function training. The total effective rate, Mini-Mental State Examination (MMSE), Montreal Cognitive Assessment (MoCA), activities of daily living (ADL), P300 peak latency, P300 amplitude, Barthel Index (BI), and Loewenstein Occupational Therapy Cognitive Assessment (LOTCA) were recorded in SRs at least once.

### 2.4. Exclusion criteria

If an SR met any of the following criteria, it was disqualified: the diagnostic criteria were unclear; the intervention was mainly non-acupuncture, or the control group received the same acupuncture therapy as the experimental group; repeated publications; inability to obtain the full text or incomplete data presented; the study was another type of research, such as animal experiments, protocols, conference papers, and case reports.

### 2.5. Study selection and data extraction

Two reviewers separately performed literature retrieval and screening in accordance with the retrieval approach. A third reviewer was contacted if there was dispute. Two researchers independently retrieved pertinent data from eligible studies using standardized extraction tables, including the author, publication year, sample size, diagnostic standards, interventions, results, adverse reactions, and conclusions. The extracted material was cross-checked by 2 reviewers who then consulted a third reviewer to resolve any disagreements.

### 2.6. Quality assessment

The Cochrane Risk Assessment Scale was used to evaluate the risk of bias in the included studies. The scale consists of 7 components: random sequence generation, allocation concealment, blinding of investigators and subjects, blinded assessment of study results, completeness of outcome data, selective reporting of study results, and other bias. Low risk of bias, high risk of bias, and uncertain risk of bias were used to categorize the risk categories.

### 2.7. Statistical analysis

Stata 17.0 was used for the analysis. The effect sizes were assessed using OR, and efficiency was a dichotomous variable. The pre- and posttreatment difference values served as effect sizes, although the MMSE, ADL (using the BI Score to Assess ADL), and MoCA scores were continuous variables.

Ratio ratios (OR) were used for dichotomous variables, and mean difference (MD) was used as a measure for continuous variables, and each effect size was given its point estimate and 95% CI.

The Bayesian framework was applied to NMA in State 17.0, utilizing the network and mvmeta packages. The study was divided into paired 2-armed tests to determine whether it was a 3-armed or more than a 3-armed trial. Evidence network maps show how several interventions are related to 1 another in direct or indirect comparisons. The dots in the graph represent interventions, and larger dots indicate that the intervention was being used by more patients. If the line was straight, there was a direct comparison between the 2 interventions. The number of studies with direct comparisons was indicated by the thickness of the line segment. The ranking probability was represented by the surface under the cumulative ranking curve (SUCRA) (0% SUCRA 100%). A higher SUCRA for an intervention denotes greater effectiveness of the intervention. We used the STATA tool to determine the inconsistency factor of the closed loop, and the 95% confidence interval of the IF to assess the loop’s consistency. Direct and indirect evidence are consistent for closed loops if the IF value of the 95% CI includes 0. Otherwise, the likelihood of inconsistency increases. To establish whether the study had a small sample effect, we also created “comparison-corrected” funnel plots using the STATA tool.

## 3. Results

### 3.1. Study selection

**Figure 1.**
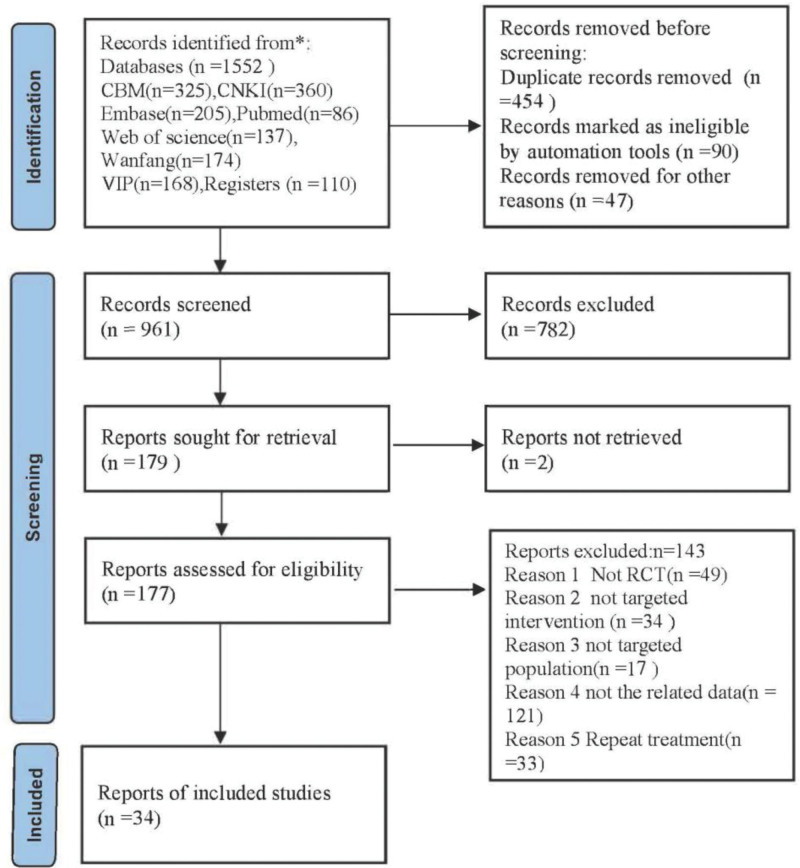
Screening process of literature selection.

A total of 1662 studies were included in the current study. The screening resulted in the removal of 591 records. A total of 177 papers were selected for additional examination after their titles and abstracts were examined. Of these, 143 were disqualified, including 33 repeat treatments, 34 targeted interventions that were not RCTs, and 17 that did not include the pertinent target populations. 34 RCTs^[16–49]^ in all were included in this meta-analysis after meeting the inclusion criteria. Five were in English and 29 were in Chinese. Both 2-armed tests were. The literature selection process is shown in Figure 1. About 2690 patients were included in the 34 studies. The essential characteristics of the included studies are presented in Table 1.

**Table 1:** Characteristics of included studies.

Study	Type of disease	Age (yr) (treatment/control)		Sample (men)	Intervention (treatment/control)	Duration of intervention	Outcome measure
Zhang 2018	Mild cognitive impairment after stroke	71 ± 2.74	71.47 ± 2.83	40 (19)/40 (20)	CT + CFT + HPN/CT + CFT + NIM	8 wk	MoCA/MMSE/ADL
Wu 2019	Cognitive impairment after stroke	64.05 ± 10.41	62.98 ± 11.41	40 (16)/40 (10)	CT + HPN/CT	4 wk	MoCA/MMSE/ADL/General effectiveness
Yang 2019	Cognitive impairment after stroke	51.35 ± 7.30	51.72 ± 7.46	40 (24)/40 (22)	CT + CFT + ACU/CT + CFT	4 wk	MMSE/ADL/General effectiveness
Liu 2013	Cognitive impairment after stroke	Unavailable	Unavailable	Unavailable	CT + CFT + EA/CT + CFT	4 wk	MMSE/ADL
Feng 2014	Cognitive impairment after stroke	64 ± 6	72 ± 8	30 (16)/30 (21)	CT + CFT + ACU/CT + CFT	8 wk	MoCA/ADL
Zhang 2016	Cognitive impairment after stroke	44 ± 4	46 ± 5	71 (35)/73 (41)	CT + AP/CT	4 wk	MMSE/ADL
Gao 2019	Cognitive impairment after stroke	67.93 ± 4.26	68.73 ± 3.24	30 (9)/30 (8)	CT + CFT + AP/CT + CFT	12 wk	MoCA/MMSE/General effectiveness
Zhang 2018	Cognitive impairment after stroke	69.5 ± 5.5	68.9 ± 4.9	38 (24)/38 (26)	CT + IDE + HPN/CT + IDE	8 wk	MMSE/ADL
Zheng 2019	Cognitive impairment after cerebral infarction	63 ± 6	67 ± 7	29 (18)/28 (19)	CT + CFT + MOX/CT + CFT	12 wk	MoCA/ADL/P300
Yao 2020	Cognitive impairment after stroke	57.4 ± 12.8	54.6 ± 11.8	30 (19)/30 (21)	CT + CFT + HPN/CT + CFT	4 wk	MoCA/MMSE/P300
Cao 2019	Cognitive impairment after stroke	60 ± 3	59 ± 3	51 (26)/49 (25)	CT + CFT + HPN/CT + CFT	6 wk	MoCA/MMSE/ADL/General effectiveness
Yan 2016	Mild cognitive impairment after ischemic stroke	65	66	30 (18)/30 (17)	CT + CFT + MOX/CT + CFT	8 wk	MoCA/MMSE/ADL
Yuan 2022	Mild cognitive impairment after stroke	61 ± 8	59 ± 9	39 (31)/40 (29)	CFT + ACU + MOX/CFT	4 wk	MoCA/MMSE/ADL
Chen 2019	Cognitive impairment after stroke	63.23 ± 7.15	62.7 ± 7.18	40 (19)/40 (17)	CT + HPN + AP/CT	4 wk	MoCA/MMSE/ADL
Niu 2021	Cognitive impairment after cerebral infarction	51.89 ± 10.24	52.06 ± 7.98	75 (41)/75 (40)	CT + CFT + HPN/CT + CFT	6 wk	MoCA/MMSE/ADL/General effectiveness
Li 2019	Mild cognitive impairment after stroke	63.17 ± 7.84	62.61 ± 7.76	34 (20)/34 (19)	CT + HPN/CT	12 wk	MMSE/ADL/General effectiveness
Zhang 2021	Mild cognitive impairment after stroke	61 ± 9	60 ± 8	25 (19)/25 (18)	CT + CFT + HPN/CT + CFT	4 wk	MoCA/MMSE/ADL
Sheng 2013	Poststroke dementia	71.83 ± 2.69	67.27 ± 1.92	30 (22)/30 (19)	CT + HPN/CT	4 wk	MMSE/ADL/General effectiveness
Chen 2020	Cognitive impairment after stroke	56.85 ± 17.12	56.95 ± 18.09	40 (22)/40 (21)	CT + CFT + HPN/CT + CFT	4 wk	MoCA/MMSE/ADL
Deng 2021	Cognitive impairment after stroke	59 ± 3	60 ± 3	40 (21)/40 (20)	CT + HPN + BLT/CT	8 wk	MoCA/MMSE/ADL/General effectiveness
Wang 2015	Cognitive impairment after stroke	46.89 ± 6.10	44.44 ± 9.92	38 (24)/38 (25)	CT + CFT + NWM/CT + CFT	4 wk	MoCA/ADL
Fang 2019	Mild cognitive impairment after stroke	65.18 ± 4.29	65.07 ± 4.31	41 (23)/41 (21)	CT + CFT + NWM/CT + CFT	6 wk	MoCA/MMSE/ADL/General effectiveness
Wang 2018	Mild cognitive impairment after stroke	71.42 ± 8.67	69.33 ± 7.56	64 (33)/64 (29)	CT + ACU/CT	10 wk	MoCA/MMSE
Gao 2019	Mild cognitive impairment after stroke	64.16 ± 7.42	65.81 ± 9.94	43 (20)/43 (24)	CT + CFT + EAT/CT + CFT	8 wk	MoCA/MMSE/ADL/General effectiveness/LOTCA
Xue 2016	Cognitive dysfunction after ischemic stroke	63 ± 7	62 ± 8	30 (19)/30 (17)	CT + CFT + HPN/CT + CFT	12 wk	MoCA/General effectiveness
Wang 2019	Mild cognitive impairment in geriatric ischemic stroke	68.88 ± 3.64	67.71 ± 3.02	59 (36)/59 (32)	CT + ACU/CT	4 wk	General effectiveness
Li 2019	Cognitive impairment after stroke	62.14 ± 8.22	57.22 ± 8.26	31 (18)/31 (20)	CT + CFT + ACU/CT + CFT	8 wk	MoCA/ADL
Wei 2019	Cognitive impairment after cerebral infarction	60.32 ± 7.93	60.38 ± 8.01	30 (18)/30 (19)	CT + CFT + ACU/CT + CFT	6 wk	MoCA/ADL
Bao 2012	Mild cognitive impairment after cerebral infarction	63 ± 6	64 ± 6	30 (19)/30 (21)	CT + ACU/CT	8 wk	MMSE/ADL/General effectiveness
Li 2012	Mild cognitive impairment after stroke	68.29 ± 8.22	69.22 ± 7.88	48 (30)/48 (24)	CT + ACU/CT	12 wk	MMSE/ADL/General effectiveness
Du 2018	Cognitive disorder after cerebral injury	40.63 ± 5.68	37 ± 7.25	30 (20)/30 (19)	CT + CFT + HPN/CT + CFT	12 wk	LOTCA
Yang 2022	Poststroke congitive impairment	65.5 ± 14	64.6 ± 13	35 (19)/35 (17)	CT + CFT + ACU/CT + CFT	8 wk	MoCA/MMSE/General effectiveness/P300
Xiao 2021	Poststroke patients	62.4 ± 3.93	61.6 ± 4.75	72 (31)/72 (34)	CT + ACU/CT	8 wk	MMSE
Wang 2016	Mild cognitive impairment after cerebral	60.6 ± 6.7	64.4 ± 7.7	42 (26)/42 (30)	ACU/NIM	12 wk	MoCA

### 3.2. Quality evaluation

**Figure 2.**
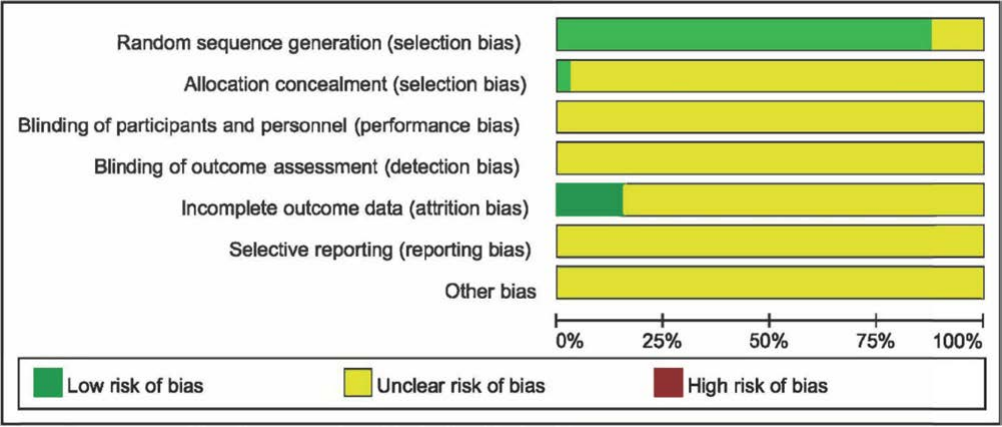
Risk of bias graph.

Of the 34 studies, 29 used the number table method to generate random sequences, 1 study^[48]^ used central randomized groups, and 1 study used parallel randomization. They were all rated as low risk. Unknown risks were assigned to 3 studies^[20,23,40]^ that stated random grouping but did not clarify how the random sequence was constructed. All other studies were assessed as unknown risk because they did not state whether allocation concealment was used; however, 1 study^[17]^ accounted for allocation concealment and was thus classified as low risk. None of the analyses were conducted with participants, implementers, or hidden results. Five studies^[17,19,21,23,41]^ provided detailed information on the frequency and reasons for shedding; the proportion missing was not enough to have an impact on the outcome of the anticipated intervention; therefore, it was assessed as low risk; the remaining studies were rated as uncertain risk. None of the studies indicated clinical study registration information, the original study protocol was unavailable, and it was not possible to determine if there was selective reporting; all were rated as unknown risks. The quality of the included studies is shown in Figure 2.

### 3.3. Network meta-analysis

#### 3.3.1. MMSE

**Figure 3.**
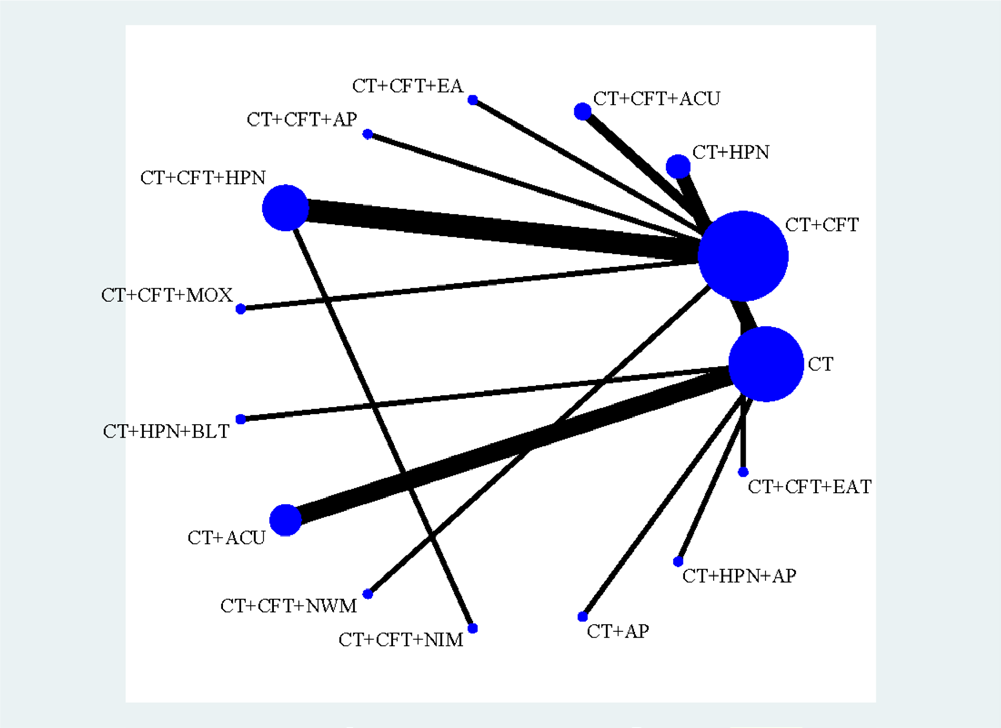
Network plot of MMSE.

In 25 studies,^[16–19,23,24,26–28,30–33,35,38–40,42,49]^ MMSE scores were published along with 15 therapies. A network plot is shown in Figure 3. No closed loop was created because all of them were immediately comparable. Hence, inconsistency testing is not required.

The NMA results show that there is no statistically significant difference between the 15 therapies used to improve MMSE scores and enhance cognitive function. (Appendix 2, Supplemental Digital Content, http://links.lww.com/MD/N786).

**Figure 4.**
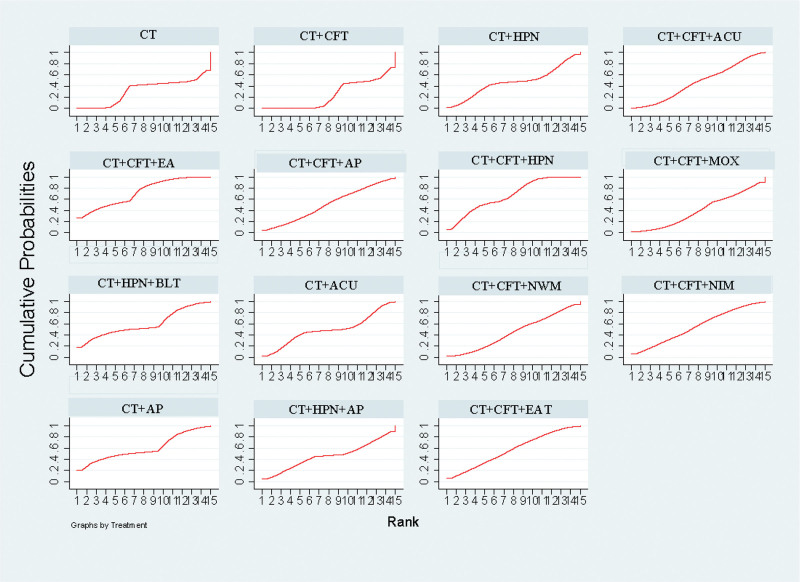
MMSE SUGAR sorting diagram.

The various acupuncture therapies that increase MMSE effectiveness SUCRA received a: CT + CFT + EA (SUCRA = 72.4%) > CT + CFT + MOX (SUCRA = 67.5%) > CT + AP (SUCRA = 59.8%) > CT + HPN + BLT (SUCRA = 59.1%) > CT + CFT + EAT (SUCRA = 56.6%) > CT + CFT + NIM (SUCRA = 55.2%) > CT + CFT + AP (SUCRA = 50.8%) > CT + ACU (SUCRA = 49%)＞ CT + HPN (SUCRA = 47%) > CT + CFT + ACU (SUCRA = 46.6%) > CT + HPN + AP (SUCRA = 45.8%) > CT + CFT + NWM (SUCRA = 44.6%) > CT + CFT + MOX (SUCRA = 40%) > CT (SUCRA = 31.6%) > CT + CFT (SUCRA = 21.4%) (Fig. 4).

#### 3.3.2. MoCA

**Figure 5.**
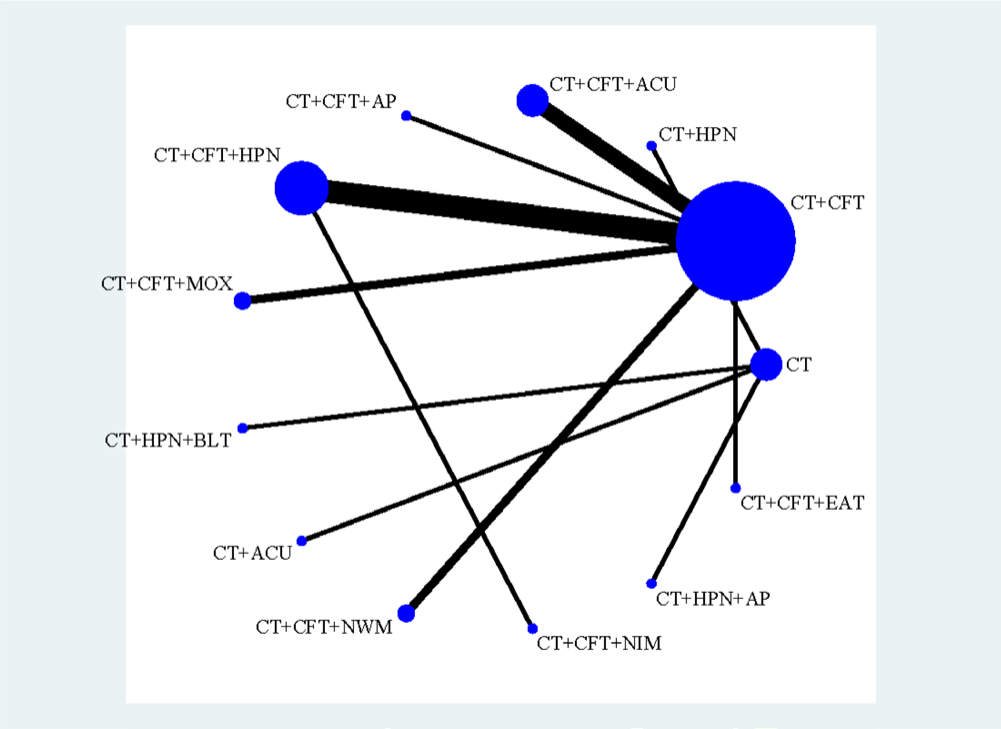
Network plot of MoCA.

Thirteen treatments were covered in 23 trials^[20–22,24–27,31–35,37–39,41–46,48,49]^ that produced MoCA scores. In Figure 5, the network plot is shown. No closed loop was created because all of them were immediately comparable. Hence, inconsistency testing is not required.

According to the NMA results, CT + CFT + NIM treatment more effectively improved MoCA than CT + CFT + EAT treatment [MD = 0.00, 95% CI (0.00, 0.35)]. None of the remaining comparable differences was statistically significant (Appendix 3, Supplemental Digital Content, http://links.lww.com/MD/N786).

**Figure 6.**
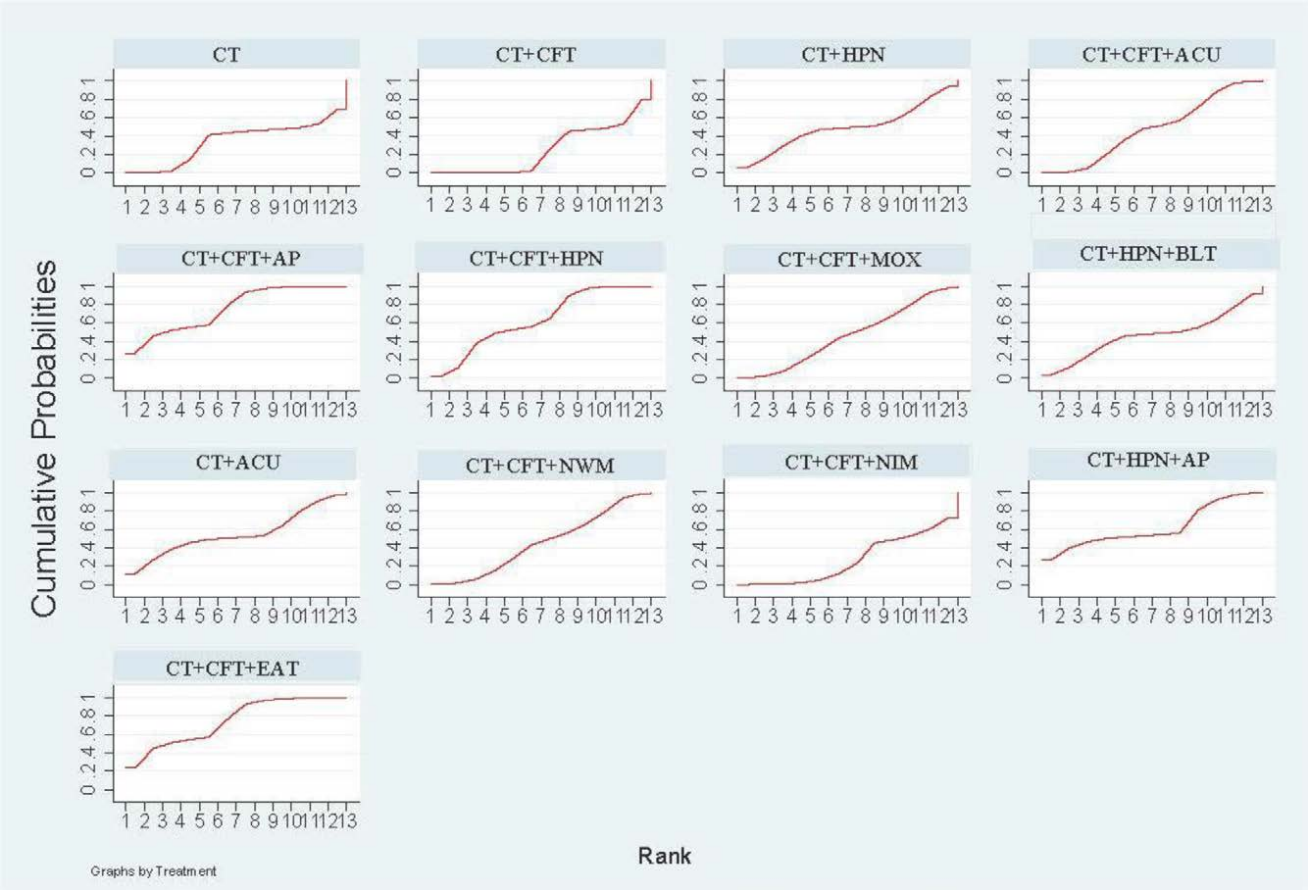
MOCA SUGAR sorting diagram.

The various acupuncture treatments to increase MoCA effectiveness SUCRA received a: CT + CFT + AP (SUCRA = 75.4%) > CT + CFT + EAT (SUCRA = 74.6%) > CT + CFT + HPN (SUCRA = 63.3%) > CT + HPN + AP (SUCRA = 62.2%) > CT + ACU (SUCRA = 54.9%) > CT + HPN (SUCRA = 48.7%) > CT + CFT + ACU (SUCRA = 47.9%) > CT + HPN + BLT (SUCRA = 46.3%) > CT + CFT + MOX (SUCRA = 45.7%) > CT + CFT + NWM (SUCRA = 45.0%) > CT (SUCRA = 34.3%) > CT + CFT + NIM (SUCRA = 26.6%) > CT + CFT (SUCRA = 25.2%) (Fig. 6).

#### 3.3.3. Activities of daily living

**Figure 7.**
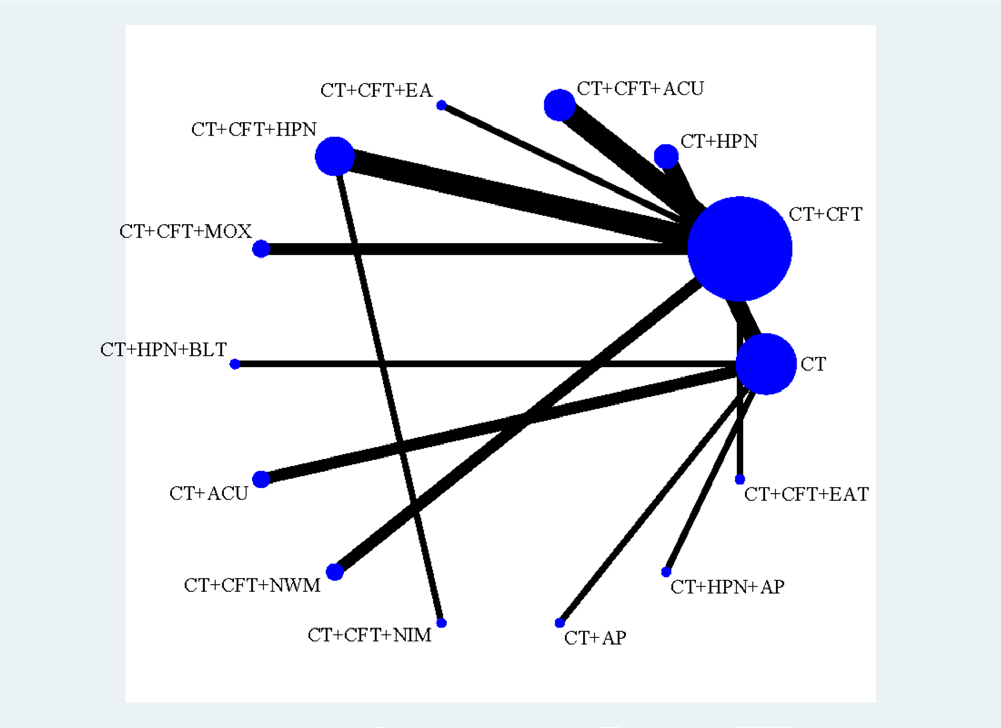
Network plot of ADL.

About 25 studies^[16–21,23,24,27,28,30–35,37,38,40–42,44–46,48]^ used the BI to record changes in ADL scores. A network plot is shown in Figure 7. No closed loop was created because all of them were immediately comparable. Hence, inconsistency testing is not required.

According to the NMA results, CT + CFT + ACU treatment more effectively improved ADL than CT + CFT + NIM treatment [MD = 1.34e+06, 95% CI (2.92, 6.16e+11)]. The effectiveness of CT + CFT + HPN and CT + CFT + HPN was higher than that of CT + CFT + NIM [MD = 69,006.39, 95% CI (1.97, 2.41e+09)], that of CT + HPN + BLT was higher than that of CT + AP [MD = 3.70e+08, 95% CI (92.96, 1.48e+15)], and that of CT + ACU was higher than that of CT + AP [MD = 6.80e+06, 95% CI (11.17, 4.14e+12)] (Appendix 4, Supplemental Digital Content, http://links.lww.com/MD/N786).

**Figure 8.**
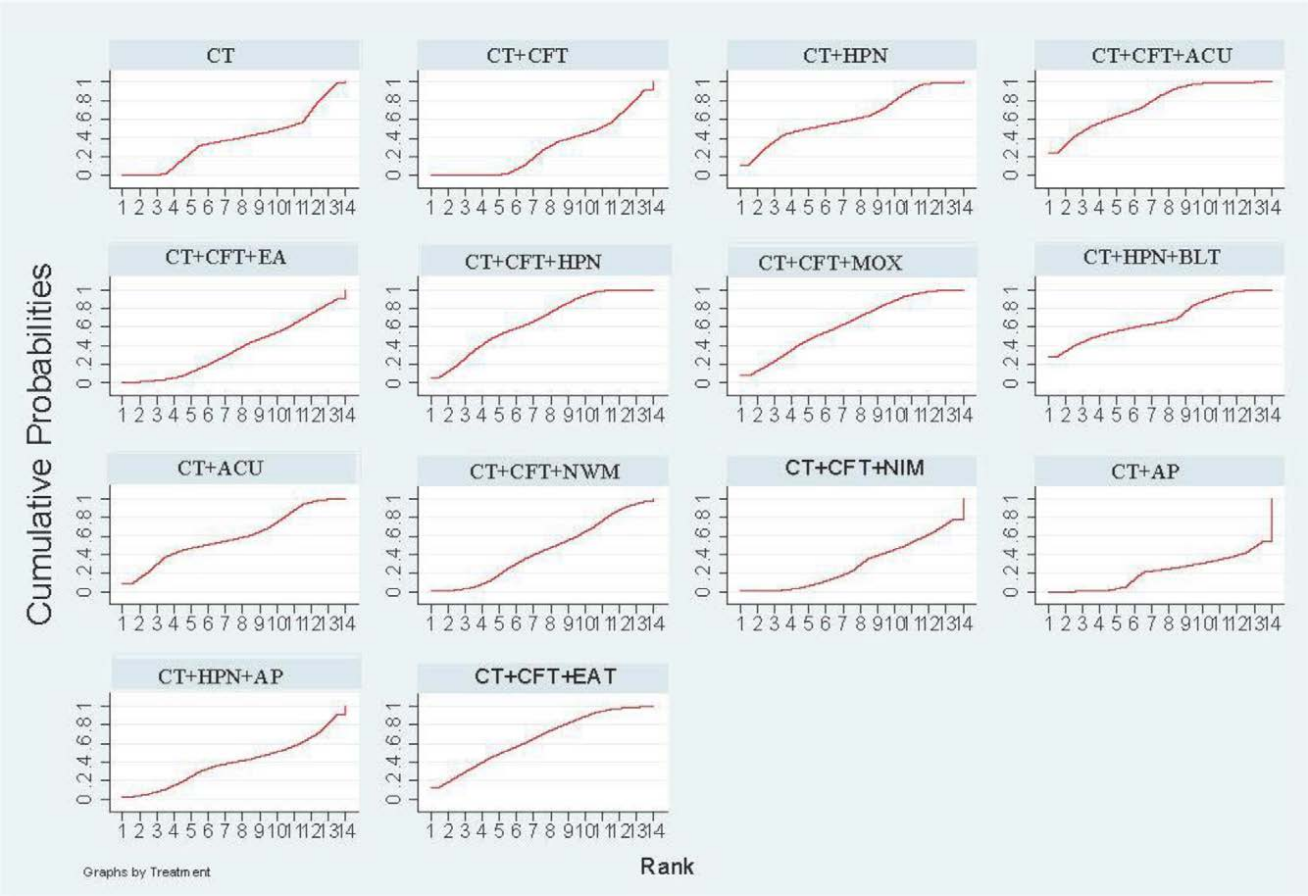
ADL SUGAR sorting diagram.

The ranking results showed that CT + CFT + ACU (SUCRA = 76.3%) was the most successful intervention for raising ADL scores, while CT + CFT (SUCRA = 29.7%) was the least successful (Fig. 8).

#### 3.3.4. Clinical efficiency

**Figure 9.**
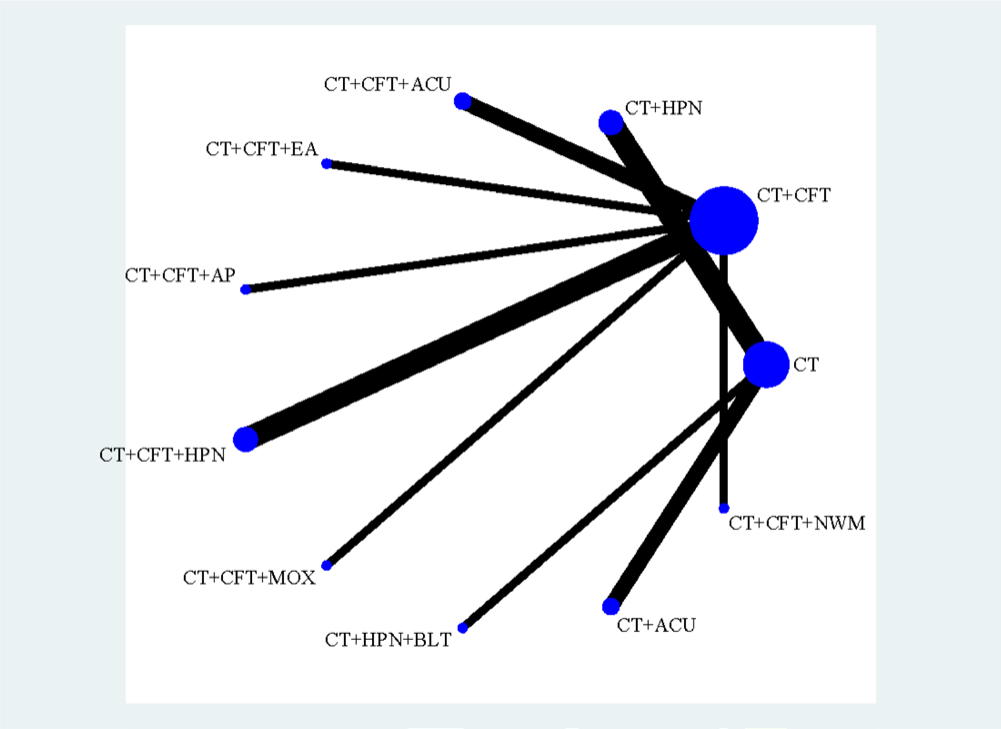
Network plot of clinical efficiency.

Clinical efficacy was reported in 16 studies,^[17–19,22,24,28,30,31,33,36,38–40,44,45,49]^ 11 therapies were included. In Figure 9, the network plot is displayed. Because they are all immediately comparable, no closed loop is created. Therefore, inconsistency testing is not required.

The NMA results demonstrated that every treatment measure improved clinical effectiveness without a statistically significant difference (Appendix 5, Supplemental Digital Content, http://links.lww.com/MD/N786).

**Figure 10.**
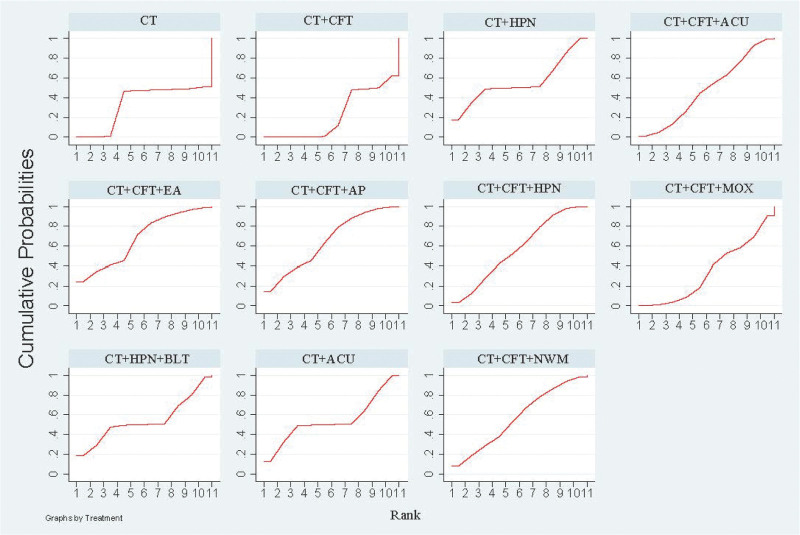
Clinical efficiency SUGAR sorting diagram.

The ranking results revealed that CT + CFT + EA (SUCRA = 68.0%) was the most successful intervention to increase clinical effectiveness, whereas CT + CFT (SUCRA = 22.2%) was the least successful (Fig. 10).

### 3.4. Adverse reaction

According to 4 studies,^[30,33,36,44]^ a few individuals develop diarrhea, dizziness, indigestion, nausea, vomiting, and sleepiness while receiving medication, according to 4 studies.^[30,33,36,44]^ After resting, the patients felt better and the therapy was unaffected. No negative effects were observed in other investigations.

### 3.5. Publication bias

**Figure 11.**
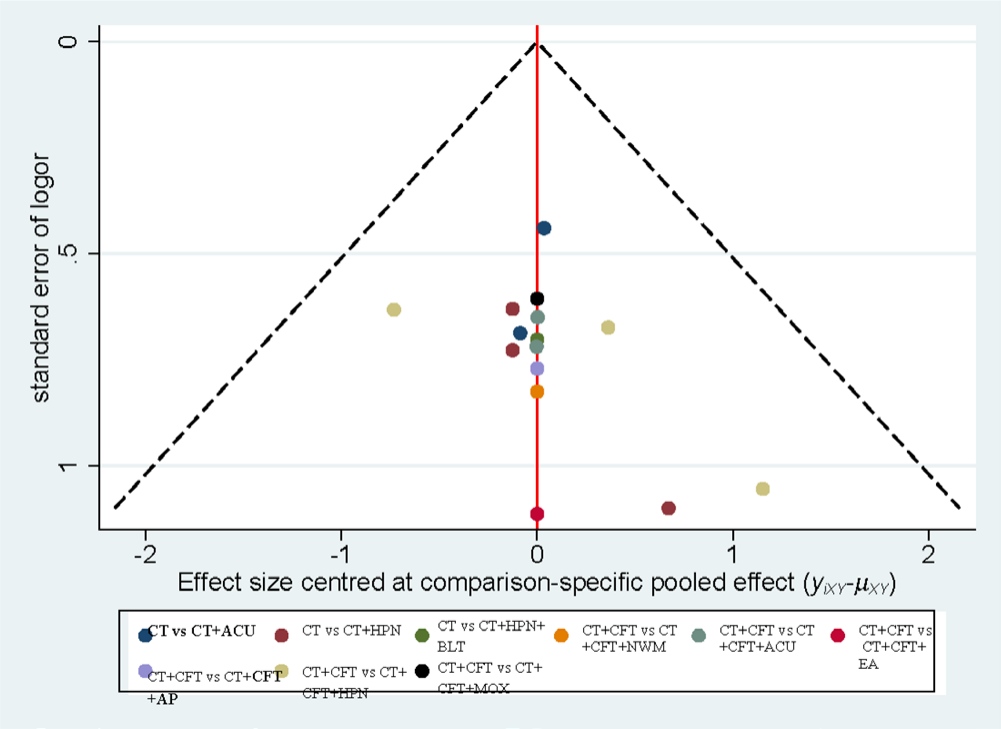
Funnel plot.

All studies were symmetrically distributed around the vertical *X* = 0 line, and the funnel plot was symmetric, indicating no evidence of minor sample effects in the study network (Fig. 11).

## 4. Discussion

To the best of our knowledge, this is the first NMA to investigate the effectiveness of various acupuncture therapies for PSCID. PSCID is described as any cognitive function issue that develops after a stroke, regardless of the cause of the stroke.^[50]^ Therefore, cognitive dysfunction is a key symptom of PSCID. The included studies used the MMSE and MoCA to measure patients’ cognitive performance, while ADL and clinical efficacy measured their capacity for daily living.

NMA revealed that both CT + CFT + ACU and CT + CFT + EA appeared to be more beneficial for daily living activities, while CT + CFT + EA and CT + CFT + AP appeared to be more helpful for cognitive performance in patients with PSCID. The findings of this study provide a theoretical foundation for the therapeutic benefits of combining acupuncture with conventional therapy for PSCID by demonstrating that various acupuncture techniques might enhance cognitive function and daily living abilities of patients with PSCID.

Acupuncture is the most prevalent type of external therapy in Traditional Chinese Medicine and is characterized by ease of use, general conditioning, quick results, a variety of activities, and no negative side effects. It features a simple technique, whole-body conditioning, quick growth, large range of motion, and no adverse effects.

Scalp acupuncture, body acupuncture, electro-acupuncture, moxibustion, and the use of acupuncture in conjunction with other treatments are just a few of the many advancements in Traditional Chinese Medicine. Acupuncture has been demonstrated to enhance cognitive function in stroke patients in recent years according to an increasing number of important clinical studies.^[14,51]^ Recent systematic reviews and meta-analyses of evidence-based medical literature suggest that acupuncture can effectively enhance cognitive function in stroke patients^[15,52,53]^ with a high safety.^[54,55]^

Acupuncture improves cognitive dysfunction primarily by improving the function of the central cholinergic system, affecting monoamine neurotransmitter release, reducing the toxicity of excitatory amino acids, and reducing inflammatory responses. Acupuncture has been shown to reduce the deposition of Aβ1-42, reduce cell damage, and promote nerve regeneration.^[56]^ Head penetration needling can adjust the function of the corresponding lobes of the brain through the projection of the cerebral cortex in the head, increase local blood flow, open the collateral circulation of the blood vessels in the brain, and improve the ischemia and hypoxia of brain tissues around the stroke foci through the alteration of serum vascular endothelial growth factor, plasma endothelin, calcitonin gene-related peptide, etc, so as to achieve the improvement of memory and other cognitive functions. The mechanism of action of electro-acupuncture to promote cognition is related to its enhancement of free radical scavenging ability, inhibition of free radical reactions in the brain, and antagonism of apoptosis in brain tissue. Improved cognition with auricular acupressure or auricular acupuncture was associated with a significant increase in hippocampal choline acetyltransferase-immunoreactive neurons after acupuncture. Hippocampal choline acetyltransferase is a key enzyme that catalyzes the synthesis of acetylcholine in the cell, which is the 1 neurotransmitter that has so far been found to have a close relationship with learning and memory functions.

## 5. Limitations

This research has certain limitations. First, NMA is an extension of conventional meta-analysis, which addresses the dearth of directly comparable clinical research data by indirectly comparing the efficacy of 3 or more therapies. The included studies’ differences in clinical characteristics influencing outcomes, such as patients, treatments, and control implementation, may have a direct impact on the conclusions and dependability of the meta-analysis, because the NMA technique is based on the assumption of homogeneity. Second, the same therapies may have other improvements in other outcome indicators; hence, the amount of clinical evidence would be reduced. NMA was performed to analyze several outcome indicators (MMSE, MoCA, and ADL scores). Interventions with greater treatment levels are advised to improve the clinical efficacy in accordance with other clinical manifestations and treatment objectives. Third, the final included literature was of low quality, and the risk bias assessment results were mostly unclear. To ensure the caliber of the literature, future studies should be planned, carried out, and reported strictly in accordance with the requirements for randomized controlled trials and the reporting requirements for randomized controlled clinical studies-CONSORT statement). Fourth, because all of the included publications are recent efficacy findings, the results only serve as an evaluation of the recent efficacy of acupuncture in patients with PSCID. Further studies are required to determine the durability of acupuncture. Fifth, there are few pieces of material that directly compare various acupuncture-related therapies. The differences and benefits of diverse acupuncture therapies can be further assessed in future studies by conducting additional research on direct comparisons among various acupuncture-related therapies.

## 6. Conclusion

Combined acupuncture therapy may be an efficient and secure strategy for individuals with PSCID. The effectiveness and safety of acupuncture for PSCID should be examined in the future through high-quality, extensive, and long-term investigations.

## Author contributions

**Data curation:** Xuezheng Zhang.

**Methodology:** Manli Zhao, Zeying Wang, Lunzhong Zhang.

**Software:** Lei Huo.

**Writing – original draft:** Lei Huo, Manli Zhao.

**Writing – review & editing:** Kaili Fu.

## Supplementary Material


