# Supplementary material for: A network meta-analysis of different acupuncture therapy in the treatment of poststroke cognitive impairment and dementia
Source: Medicine (Baltimore). 2024 Oct 25;103(43):e40233. doi: 10.1097/MD.0000000000040233 (PMC11521021; doi:10.1097/MD.0000000000040233)
Supplement: Supplementary file 1 [file medi-103-e40233-s001.pdf]

Supplementary Material

A network meta-analysis of different acupuncture therapy in the treatment of Post-Stroke Cognitive Impairment and Dementia

Lei Huo and Manli Zhao are co-first authors.

*** Correspondence:** Kaili Fu: fklfw@163.com

16.1 Appendix 1 Retrieval strategy

16.1.1 Appendix 1.1 search strategy for PubMed database

NO	Search items
#1	Cognitive Dysfunction[MeSH Terms]
#2	cognitive dysfunction*[Title/Abstract]
#3	cognitive impairment*[Title/Abstract]
#4	cognitive disorder*[Title/Abstract]
#5	mild cognitive impairment*[Title/Abstract]
#6	cognitive decline*[Title/Abstract]
#7	mental deterioration*"[Title/Abstract]
#8	#1 OR #2 OR #3 OR #4 OR #5 OR #6 OR #7
#9	Stroke[MeSH Terms]
#10	stroke*[Title/Abstract]
#11	cerebrovascular accident*[Title/Abstract]
#12	Cva [All Fields] AND cerebrovascular accident[Title/Abstract]
#13	cerebrovascular apoplexy[Title/Abstract]
#14	brain vascular accident*[Title/Abstract]
#15	cerebrovascular stroke*[Title/Abstract]
#16	Apoplexy[Title/Abstract]
#17	cerebral stroke*[Title/Abstract]
#18	acute stroke*[Title/Abstract]
#19	acute cerebrovascular accident*"[Title/Abstract]
#20	#9 OR #10 OR #11 OR #12 OR #13 OR #14 OR #15 OR #16 OR #17 OR #18 OR #19
#21	Needles[MeSH Terms]
#22	needle*[Title/Abstract]
#23	Acupuncture[Title/Abstract]
#24	hypodermic needle*[Title/Abstract]
#25	fire needle[Title/Abstract]
#26	balance acupuncture[Title/Abstract]
#27	Electroacupuncture[Title/Abstract]

#28	auricular acupressure[Title/Abstract]
#29	auricular acupuncture[Title/Abstract]
#30	acupoints catgut embedding[Title/Abstract]
#31	plum blossom needle[Title/Abstract]
#32	head penetration needling[Title/Abstract]
#33	intradermal needle[Title/Abstract]
#34	acupoint application[Title/Abstract]
#35	#21 OR #22 OR #23 OR #24 OR #25 OR #26 OR #27 OR #28 OR #29 OR #30 OR #31 OR #32 OR #33 OR #34
#36	#8 AND #20 AND #35

16.1.2 **Appendix 1.2** search strategy for Web of science

NO	Search items
#1	TS=(Cognitive Dysfunction*)
#2	TS=(Cognitive Impairment*)
#3	TS=(Cognitive Disorder*)
#4	TS=(Mild Cognitive Impairment*)
#5	TS=(Cognitive Decline*)
#6	TS=(Mental Deterioration*)
#7	#1 OR #2 OR #3 OR #4 OR #5 OR #6
#8	TS=(Stroke*)
#9	TS=(Cerebrovascular Accident*)
#10	TS=(CVA* (Cerebrovascular Accident))
#11	TS=(Cerebrovascular Apoplexy)
#12	TS=(Brain Vascular Accident*)
#13	TS=(Cerebrovascular Stroke*)
#14	TS=(Apoplexy)
#15	TS=(Cerebral Stroke*)
#16	TS=(Acute Stroke*)
#17	TS=(Acute Cerebrovascular Accident*)
#18	#8 OR #10 OR #11 OR #12 OR #13 OR #14 OR #15 OR #16 OR #17
#19	TS=(Acupuncture)
#20	TS=(Needle*)
#21	TS=(Hypodermic Needle*)
#22	TS=(Fire needle)
#23	TS=(balance acupuncture)
#24	TS=(Electroacupuncture)
#25	TS=(auricular acupressure)
#26	TS=(auricular acupuncture)
#27	TS=(acupoints catgut embedding)

#28	TS=(acupoint application)
#29	TS=(plum blossom needle)
#30	TS=(head penetration needling)
#31	TS=(intradermal needle)
#32	#19 OR #20 OR #21 OR #22 OR #23 OR #24 OR #25 OR #26 OR #27 OR #28 OR #29 OR #30 OR #31
#33	#7 AND #18 AND #32

16.1.3 **Appendix 1.3** search strategy for CNKI

NO	Search items
#1	Cognitive Impairment*
#2	mental and behavior disorder
#3	dementia
#4	#1 OR #2 OR #3
#5	cerebral arterial thrombosis
#6	Stroke
#7	#5 OR #6
#8	needle*
#9	Acupuncture
#10	warm acupuncture
#11	Electroacupuncture
#12	Fire needle
#13	balance acupuncture
#14	auricular acupressure
#15	auricular acupuncture
#16	Hypodermic Needle*
#17	acupoints catgut embedding
#18	acupoint application
#19	plum blossom needle
#20	head penetration needling
#21	intradermal needle
#22	#8 OR #9 OR #10 OR #11 OR #12 OR #13 OR #14 OR #15 OR #16 OR #17 OR #18 OR #19 OR #20 OR #21
#23	Random*
#24	#4 AND #7 AND #22 AND #23

Supplementary Material

A network meta-analysis of different acupuncture therapy in the treatment of Post-Stroke Cognitive Impairment and Dementia

Lei Huo and Manli Zhao are co-first authors.

*** Correspondence:** Kaili Fu: fklfw@163.com

16.2 **Appendix 2** Network meta-analysis of MMSE improved by different acupuncture treatments

CT	0.67 (0.00,2.76e+23)	6.38 (0.51,79.02)	11.46 (0.00,5.06e+24)	359.16 (0.00,1.71e+26)	17.53 (0.00,8.85e+24)	116.36(0.00, 4.87e+25)	4.24 (0.00,2.10e+24)	47.47 (0.71,3182.25)	8.08 (0.92,71.14)	8.36 (0.00,3.97e+24)	33.97 (0.00,1.64e+25)	54.60 (0.70, 4274.52)	5.42 (0.09,344.02)	36.74 (0.00,1.75e+25)
1.49 (0.00,6.11e+23)	CT+CFT	9.48 (0.00,4.13e+24)	17.04(0.83,349.38)	533.83 (8.53,33402.76)	26.05 (0.20,3428.47)	172.94(24.14,1239.05)	6.30 (0.06,658.29)	70.55 (0.00,3.41e+25)	12.00 (0.00,5.15e+24)	12.43 (0.20,781.53)	50.50 (0.53,4804.08)	81.15 (0.00, 3.97e+25)	8.06 (0.00,3.88e+24)	54.60 (0.87,3436.50)

0.16 (0.01,1.94)	0.11 (0.00,4.59e+22)	CT+HPN	1.80 (0.00,8.41e+23)	56.32 (0.00,2.84e+25)	2.75 (0.00,1.47e+24)	18.25 (0.00,8.09e+24)	0.66 (0.00,3.49e+23)	7.44 (0.06,1000.53)	1.27 (0.05,35.22)	1.31 (0.00,6.60e+23)	5.33 (0.00,2.73e+24)	8.56 (0.06,1315.51)	0.85 (0.01,109.01)	5.76 (0.00,2.90e+24)
0.09 (0.00,3.85e+22)	0.06 (0.01,1.20)	0.56 (0.00,2.60e+23)	CT+CFT+ACU	31.34 (0.19,5254.12)	1.53 (0.00,475.30)	10.15 (0.28,373.73)	0.37 (0.00,94.59)	4.14 (0.00,2.15e+24)	0.70 (0.00,3.25e+23)	0.73 (0.00,122.82)	2.96 (0.01,700.96)	4.76 (0.00,2.50e+24)	0.47 (0.00,2.44e+23)	3.21 (0.02,539.94)
0.00 (0.00,1.32e+21)	0.00 (0.00,1.12)	0.02 (0.00,8.94e+21)	0.03 (0.00,5.35)	CT+CFT+EA	0.05 (0.00,29.28)	0.32 (0.00,31.62)	0.01 (0.00,5.95)	0.13 (0.00,7.38e+22)	0.02 (0.00,1.12e+22)	0.02 (0.00,8.11)	0.09 (0.00,44.47)	0.15 (0.00,8.59e+22)	0.02 (0.00,8.39e+21)	0.10 (0.00,35.65)
0.06 (0.00,2.88e+22)	0.04 (0.00,5.05)	0.36 (0.00,1.95e+23)	0.65(0.00,203.23)	20.49 (0.03,12295.09)	CT+CFT+AP	6.64 (0.03,1280.42)	0.24 (0.00,204.43)	2.71 (0.00,1.61e+24)	0.46 (0.00,2.43e+23)	0.48 (0.00,287.18)	1.94 (0.00,1536.62)	3.11 (0.00,1.87e+24)	0.31 (0.00,1.83e+23)	2.10 (0.00,1262.33)

0.01 (0.00,3.60e+21)	0.01 (0.00,1.04)	0.05 (0.00,2.43e+22)	0.10 (0.00,3.63)	3.09 (0.03,301.33)	0.15 (0.00,29.06)	CT+CFT+HPN	0.04 (0.00,5.68)	0.41 (0.00,2.01e+23)	0.07 (0.00,3.03e+22)	0.07 (0.00,7.05)	0.29 (0.00,17.76)	0.47 (0.00, 2.34e+23)	0.05 (0.00,2.28e+22)	0.32 (0.00,30.98)
0.24 (0.00,1.17e+23)	0.16 (0.00,16.60)	1.51 (0.00,7.90e+23)	2.71(0.01,692.22)	84.77 (0.17,42749.49)	4.14 (0.00,3498.90)	27.46 (0.18,4282.01)	CT+CFT+MOX	11.20 (0.00,6.52e+24)	1.91 (0.00,9.86e+23)	1.97 (0.00,998.59)	8.02 (0.01,5382.66)	12.89 (0.00, 7.59e+24)	1.28 (0.00,7.41e+23)	8.67 (0.02,4389.53)
0.02 (0.00,1.41)	0.01 (0.00,6.85e+21)	0.13 (0.00,18.06)	0.24 (0.00,1.25e+23)	7.57 (0.00,4.23e+24)	0.37 (0.00,2.19e+23)	2.45 (0.00,1.21e+24)	0.09 (0.00,5.20e+22)	CT+HPN+BLT	0.17 (0.00,19.37)	0.18 (0.00,9.84e+22)	0.72 (0.00,4.06e+23)	1.15 (0.00, 491.72)	0.11 (0.00,42.05)	0.77 (0.00,4.32e+23)
0.12 (0.01,1.09)	0.08 (0.00,3.57e+22)	0.79 (0.03,21.96)	1.42 (0.00,6.54e+23)	44.47 (0.00,2.21e+25)	2.17 (0.00,1.14e+24)	14.41 (0.00,6.30e+24)	0.52 (0.00,2.71e+23)	5.88 (0.05,669.10)	CT+ACU	1.04 (0.00,5.14e+23)	4.21 (0.00,2.12e+24)	6.76 (0.05, 883.76)	0.67 (0.01,72.78)	4.55 (0.00,2.26e+24)

0.12 (0.00,5.68e+22)	0.08 (0.00,5.06)	0.76 (0.00,3.84e+23)	1.37(0.01,230.69)	42.95 (0.12,14958.56)	2.10 (0.00,1261.56)	13.91 (0.14,1364.21)	0.51 (0.00,256.30)	5.68 (0.00,3.17e+24)	0.97 (0.00,4.79e+23)	CT+CFT+NWM	4.06 (0.01,1916.17)	6.53 (0.00,3.69e+24)	0.65 (0.00,3.61e+23)	4.39 (0.01,1536.33)
0.03 (0.00,1.42e+22)	0.02 (0.00,1.88)	0.19 (0.00,9.60e+22)	0.34(0.00,79.77)	10.57 (0.02,4969.86)	0.52 (0.00,408.98)	3.42 (0.06,208.28)	0.12 (0.00,83.70)	1.40 (0.00,7.93e+23)	0.24 (0.00,1.20e+23)	0.25 (0.00,116.10)	CT+CFT+NIM	1.61 (0.00,9.23e+23)	0.16 (0.00,9.02e+22)	1.08 (0.00,510.33)
0.02 (0.00,1.43)	0.01 (0.00,6.03e+21)	0.12 (0.00,17.95)	0.21 (0.00,1.10e+23)	6.58 (0.00,3.72e+24)	0.32 (0.00,1.93e+23)	2.13 (0.00,1.06e+24)	0.08 (0.00,4.57e+22)	0.87 (0.00,371.63)	0.15 (0.00,19.34)	0.15 (0.00,8.66e+22)	0.62 (0.00,3.58e+23)	CT+AP	0.10 (0.00,40.86)	0.67 (0.00,3.81e+23)
0.18 (0.00,11.71)	0.12 (0.00,5.97e+22)	1.18 (0.01,150.95)	2.11 (0.00,1.09e+24)	66.27 (0.00,3.69e+25)	3.23 (0.00,1.91e+24)	21.47(0.00,1.05e+25)	0.78 (0.00,4.53e+23)	8.76 (0.02,3225.18)	1.49 (0.00,1161.63)	1.54 (0.00,8.59e+23)	6.27 (0.00,3.54e+24)	10.07 (0.02,4146.85)	CT+HPN+AP	6.78 (0.00,3.77e+24)

0.03(0.00,1.29e+22)	0.02(0.00,1.15)	0.17(0.00,8.74e+22)	0.31(0.00,52.55)	9.78(0.03,3407.42)	0.48(0.00,287.35)	3.17(0.03,310.81)	0.12(0.00,58.38)	1.29(0.00,7.22e+23)	0.22(0.00,1.09e+23)	0.23(0.00,79.61)	0.92(0.00,436.47)	1.49(0.00,8.41e+23)	0.15(0.00,8.21e+22)	CT+CFT+EAT

Supplementary Material

A network meta-analysis of different acupuncture therapy in the treatment of Post-Stroke Cognitive Impairment and Dementia

Lei Huo and Manli Zhao are co-first authors.

*** Correspondence:** Kaili Fu: fklfw@163.com

16.3 Appendix 3 Network meta-analysis of MoCA improved by different acupuncture treatments

CT	0.81 (0.00,3.61e+20)	4.39 (0.27,72.70)	6.8(0.00,3.12e+21)	155.20 (0.00,7.50e+22)	26.71 (0.00,1.20e+22)	5.66 (0.00,2.66e+21)	3.32 (0.21,53.08)	9.58 (0.53,172.85)	5.25(0.00,2.44e+21)	0.68 (0.00,3.30e+20)	26.84(1.51,476.82)	139.03(0.00,6.73e+22)
1.23 (0.00,5.44e+20)	CT+CFT	5.39 (0.00,2.60e+21)	8.45(1.67,42.83)	190.57 (10.17,3570.46)	32.80 (8.92,120.69)	6.95 (0.59,81.65)	4.08 (0.00,1.96e+21)	11.77 (0.00,5.70e+21)	6.44 (0.71,58.41)	0.84 (0.04,18.14)	32.96 (0.00,1.59e+22)	170.72 (8.71,3345.55)
0.23 (0.01,3.77)	0.19 (0.00,8.93e+19)	CT+HPN	1.57(0.00,7.72e+20)	35.33 (0.00,1.85e+22)	6.08 (0.00,2.96e+21)	1.29 (0.00,6.58e+20)	0.76 (0.01,39.03)	2.18 (0.04,122.74)	1.19 (0.00,6.03e+20)	0.15 (0.00,8.15e+19)	6.11 (0.11,340.06)	31.65 (0.00,1.67e+22)

0.15 (0.00,6.60e+19)	0.12 (0.02,1.60)	0.64 (0.00,3.15e+20)	CT+CFT+ACU	22.57 (0.79,643.29)	3.88 (0.48,31.15)	0.82 (0.04,15.76)	0.48 (0.00,2.37e+20)	1.39 (0.00,6.90e+20)	0.76 (0.05,11.84)	0.10 (0.00,3.21)	3.90 (0.00,1.93e+21)	20.21 (0.68,599.42)
0.01 (0.00,3.11e+18)	0.01 (0.00,1.10)	0.03 (0.00,1.49e+19)	0.04(0.00,1.26)	CT+CFT+AP	0.17 (0.01,4.25)	0.04 (0.00,1.68)	0.02 (0.00,1.12e+19)	0.06 (0.00,3.26e+19)	0.03 (0.00,1.32)	0.00 (0.00,0.31)	0.17 (0.00,9.11e+19)	0.90 (0.01,58.33)
0.04 (0.00,1.68e+19)	0.03 (0.01,1.11)	0.16 (0.00,8.01e+19)	0.26(0.03,2.06)	5.81 (0.24,143.52)	CT+CFT+HPN	0.21 (0.01,3.44)	0.12 (0.00,6.04e+19)	0.36 (0.00,1.76e+20)	0.20 (0.02,2.54)	0.03 (0.00,0.41)	1.00 (0.00,4.91e+20)	5.20 (0.20,133.96)
0.18 (0.00,8.32e+19)	0.14 (0.01,1.69)	0.78 (0.00,3.97e+20)	1.22(0.06,23.30)	27.43 (0.60,1262.13)	4.72 (0.29,76.67)	CT+CFT+MOX	0.59 (0.00,2.99e+20)	1.69 (0.00,8.70e+20)	0.93 (0.03,25.29)	0.12 (0.00,6.20)	4.74 (0.00,2.43e+21)	24.58 (0.52,1170.35)
0.30 (0.02,4.82)	0.25 (0.00,1.18e+20)	1.32 (0.03,68.34)	2.07 (0.00,1.02e+21)	46.75 (0.00,2.45e+22)	8.05 (0.00,3.91e+21)	1.70 (0.00,8.69e+20)	CT+HPN+BLT	2.89 (0.05,158.56)	1.58 (0.00,7.96e+20)	0.20 (0.00,1.08e+20)	8.08 (0.15,439.27)	41.88 (0.00,2.20e+22)
0.10 (0.01,1.88)	0.08 (0.00,4.11e+19)	0.46 (0.01,25.79)	0.72 (0.00,3.56e+20)	16.20 (0.00,8.54e+21)	2.79 (0.00,1.36e+21)	0.59 (0.00,3.03e+20)	0.35 (0.01,19.03)	CT+ACU	0.55 (0.00,2.78e+20)	0.07 (0.00,3.75e+19)	2.80 (0.05,165.62)	14.51 (0.00,7.67e+21)

0.19 (0.00,8.85e+19)	0.16 (0.02,1.41)	0.84 (0.00,4.22e+20)	1.31(0.08,20.33)	29.57 (0.76,1156.97)	5.09 (0.39,65.85)	1.08 (0.04,29.38)	0.63 (0.00,3.19e+20)	1.83 (0.00,9.26e+20)	CT+CFT+NWM	0.13 (0.00,5.71)	5.11 (0.00, 2.59e+21)	26.49 (0.65,1074.46)
1.47 (0.00,7.12e+20)	1.20 (0.06,25.99)	6.46 (0.00,3.40e+21)	10.11 (0.31,328.13)	228.11 (3.25,15987.04)	39.26 (2.42,638.32)	8.32 (0.16,428.68)	4.88 (0.00,2.56e+21)	14.09 (0.00,7.45e+21)	7.71 (0.18,339.90)	CT+CFT+NIM	39.45 (0.00, 2.08e+22)	204.35 (2.83,14774.29)
0.04 (0.00,1.66)	0.03 (0.00,1.47e+19)	0.16 (0.00,9.11)	0.26 (0.00,1.27e+20)	5.78 (0.00,3.05e+21)	1.00 (0.00,4.87e+20)	0.21 (0.00,1.08e+20)	0.12 (0.00,6.72)	0.36 (0.01,21.11)	0.20 (0.00,9.91e+19)	0.03 (0.00,1.34e+19)	CT+HPN+AP	5.18 (0.00,2.74e+21)
0.01 (0.00,3.48e+18)	0.01 (0.00,1.11)	0.03 (0.00,1.66e+19)	0.05(0.00,1.47)	1.12 (0.02,72.69)	0.19 (0.01,4.95)	0.04 (0.00,1.94)	0.02 (0.00,1.25e+19)	0.07 (0.00,3.65e+19)	0.04 (0.00,1.53)	0.00 (0.00,0.35)	0.19 (0.00, 1.02e+20)	CT+CFT+EAT

Supplementary Material

A network meta-analysis of different acupuncture therapy in the treatment of Post-Stroke Cognitive Impairment and Dementia

Lei Huo and Manli Zhao are co-first authors.

*** Correspondence:** Kaili Fu: fklfw@163.com

16.4 Appendix 4 Network meta-analysis of ADL improved by different acupuncture treatments

CT	0.52 (0.00,5.29e+21)	3091.48 (5.12,1.87e+06)	195652.12 (0.00,2.49e+27)	2.28 (0.00,5.86e+22)	10061.77 (0.00,1.26e+26)	4484.1(0.00,8.88e+25)	45706.67 (0.81,2.59e+09)	839.00 (0.25,2.87e+06)	23.9 (0.00,4.04e+23)	0.15 (0.00,4.95e+21)	0.00 (0.00,4.68)	0.53 (0.00,515660.34)	11309.35(0.00,3.05e+26)
1.93 (0.00,1.97e+22)	CT +CFT	5966.23 (0.00,9.11e+25)	377588.90(1720.70,8.29e+07)	4.40 (0.00,102530.23)	19418.20 (71.92,5.24e+06)	8653.97 (1.56,4.79e+07)	88209.27 (0.00,2.90e+27)	1619.18 (0.00,3.16e+25)	46.23(0.02,92179.58)	0.28 (0.00,39955.37)	0.00 (0.00,7.20e+18)	1.03 (0.00,6.61e+22)	21825.89 (0.73,6.50e+08)
0.00 (0.00,0.20)	0.00 (0.00,2.56e+18)	CT +HPN	63.29 (0.00,1.20e+24)	0.00 (0.00,2.81e+19)	3.25 (0.00,6.09e+22)	1.45 (0.00,4.28e+22)	14.78 (0.00,4.75e+06)	0.27 (0.00,8532.42)	0.01 (0.00,1.95e+20)	0.00 (0.00,2.38e+18)	0.00 (0.00,0.01)	0.00 (0.00,686.56)	3.66 (0.00,1.47e+23)

0.00 (0.00,6.51e+16)	0.00 (0.00,1.00)	0.02 (0.00,3.00e+20)	CT+CFT+ACU	0.00 (0.00,1.05)	0.05 (0.00,123.23)	0.02 (0.00,599.25)	0.23 (0.00,9.52e+21)	0.00 (0.00,1.04e+20)	0.00 (0.00,1.36)	0.00 (0.00,0.34)	0.00 (0.00,2.37e+13)	0.00 (0.00,2.17e+17)	0.06 (0.00,6478.87)
0.44 (0.00,1.13e+22)	0.23 (0.00,5303.95)	1356.98 (0.00,5.18e+25)	85880.17 (0.95,7.75e+09)	CT+CFT+EA	4416.55 (0.04,4.40e+08)	1968.29 (0.01,1.11e+09)	20062.63 (0.01,1.63e+27)	368.27 (0.01,1.79e+25)	10.51 (0.03,3.13e+06)	0.06 (0.00,363371.82)	0.00 (0.00,4.05e+18)	0.23 (0.00,3.67e+22)	4964.16 (0.00,8.88e+09)
0.00 (0.00,1.24e+18)	0.00 (0.00,1.01)	0.31 (0.00,5.75e+21)	19.45 (0.01,46594.31)	0.00 (0.00,22.57)	CT+CFT+HPN	0.45 (0.00,12766.46)	4.54 (0.00,1.82e+23)	0.08 (0.00,1.99e+21)	0.00 (0.00,29.70)	0.00 (0.00,0.51)	0.00 (0.00,4.52e+14)	0.00 (0.00,4.14e+18)	1.12 (0.00,138871.51)
0.00 (0.00,4.42e+18)	0.00 (0.00,1.64)	0.69 (0.00,2.04e+22)	43.63 (0.00,1.14e+06)	0.00 (0.00,287.26)	2.24 (0.00,64277.29)	CT+CFT+MOX	10.19 (0.00,6.40e+23)	0.19 (0.00,7.03e+21)	0.01 (0.00,520.68)	0.00 (0.00,75.13)	0.00 (0.00,1.59e+15)	0.00 (0.00,1.45e+19)	2.52 (0.00,1.72e+06)
0.00 (0.00,1.24)	0.00 (0.00,3.72e+17)	0.07 (0.00,21744.96)	4.28 (0.00,1.74e+23)	0.00 (0.00,4.04e+18)	0.22 (0.00,8.81e+21)	0.1(0.00,6.16e+21)	CT+HPN+BLT	0.02 (0.00,15380.64)	0.00 (0.00,2.81e+19)	0.00 (0.00,3.39e+17)	0.00 (0.00,0.01)	0.00 (0.00,513.04)	0.25 (0.00,2.10e+22)

0.00 (0.00,4.08)	0.00 (0.00,1.21e+19)	3.68 (0.00,115845.96)	233.20 (0.00,5.67e+24)	0.00 (0.00,1.32e+20)	11.99 (0.00,2.86e+23)	5.34 (0.00,2.01e+23)	54.48 (0.00,4.56e+07)	CT+ACU	0.03 (0.00,9.15e+20)	0.00 (0.00,1.11e+19)	0.00 (0.00,0.09)	0.00 (0.00,5675.18)	13.48 (0.00,6.88e+23)
0.04 (0.00,7.03e+20)	0.02 (0.00,43.13)	129.06 (0.00,3.24e+24)	8167.62 (0.73,9.09e+07)	0.10 (0.00,28320.25)	420.03 (0.03,5.24e+06)	187.19(0.00,1.82e+07)	1908.05 (0.00,1.02e+26)	35.02 (0.00,1.12e+24)	CT+CFT+NWM	0.01 (0.00,7957.34)	0.00 (0.00,2.54e+17)	0.02 (0.00,2.32e+21)	472.12 (0.00,1.71e+08)
6.86 (0.00,2.33e+23)	3.55 (0.00,504586.46)	21202.17 (0.00,1.07e+27)	1.34e+06 (2.92,6.16e+11)	15.62 (0.00,8.87e+07)	69006.39 (1.97,2.41e+09)	30753.59(0.01,7.11e+10)	313468.95 (0.00,3.33e+28)	5754.08 (0.00,3.68e+26)	164.29 (0.00,2.15e+08)	CT+CFT+NIM	0.00 (0.00,8.30e+19)	3.65 (0.00,7.50e+23)	77562.59 (0.01,5.17e+11)
8103.08 (0.21,3.08e+08)	4198.71 (0.00,1.27e+26)	2.51e+07 (109.96,5.71e+12)	1.59e+09 (0.00,5.95e+31)	18460.44 (0.00,1.38e+27)	8.15e+07 (0.00,3.00e+30)	3.63e+07(0.00,2.10e+30)	3.70e+08 (92.96,1.48e+15)	6.80e+06 (11.17,4.14e+12)	194106.22(0.00,9.58e+27)	1181.51 (0.00,1.16e+26)	CT+AP	4315.64 (0.00,1.48e+11)	9.16e+07 (0.00,7.18e+30)
1.88 (0.00,1.82e+06)	0.97 (0.00,6.26e+22)	5804.59 (0.00,2.31e+10)	367358.54 (0.00,2.92e+28)	4.28 (0.00,6.71e+23)	18892.09 (0.00,1.48e+27)	8419.50 (0.00,1.03e+27)	85819.34 (0.00,3.78e+12)	1575.31 (0.00,1.41e+10)	44.98 (0.00,4.69e+24)	0.27 (0.00,5.62e+22)	0.00 (0.00,7972.84)	CT+HPN+AP	21234.57 (0.00,3.49e+27)

0.00 (0.00,2.39e+18)	0.00 (0.00,1.37)	0.27 (0.00,1.10e+22)	17.30 (0.00,1.94e+06)	0.00 (0.00,360.26)	0.89 (0.00,109922.53)	0.40 (0.00,270283.05)	4.04 (0.00,3.43e+23)	0.07 (0.00,3.79e+21)	0.00 (0.00,767.57)	0.00 (0.00,85.86)	0.00 (0.00,8.54e+14)	0.00 (0.00,7.75e+18)	CT+CFT+EAT

Supplementary Material

A network meta-analysis of different acupuncture therapy in the treatment of Post-Stroke Cognitive Impairment and Dementia

Lei Huo and Manli Zhao are co-first authors.

*** Correspondence:** Kaili Fu: fklfw@163.com

16.5 Appendix 5 Network meta-analysis of Clinical efficiency improved by different acupuncture treatments

CT	1.00 (0.00, 2.36e+28)	4.37 (1.86, 10.30)	3.09 (0.00, 7.35e+28)	9.33 (0.00, 2.28e+29)	6.93 (0.00, 1.66e+29)	4.45 (0.00, 1.06e+29)	1.71 (0.00, 4.09e+28)	4.11 (1.04, 16.29)	4.15 (2.01, 8.58)	4.73(0.00,1.14e+29)
1.00 (0.00, 2.36e+28)	CT+CFT	4.37 (0.00, 1.04e+29)	3.09 (1.20, 7.95)	9.33 (1.05, 82.78)	6.93 (1.53, 31.38)	4.45 (1.95, 10.19)	1.71 (0.52, 5.62)	4.11 (0.00, 9.84e+28)	4.15 (0.00, 9.84e+28)	4.73 (0.94, 23.82)
0.23 (0.10, 1.54)	0.23 (0.00, 5.42e+27)	CT+HPN	0.71 (0.00, 1.69e+28)	2.13 (0.00, 5.25e+28)	1.58 (0.00, 3.82e+28)	1.02 (0.00, 2.43e+28)	0.39 (0.00, 9.40e+27)	0.94 (0.19, 4.76)	0.95 (0.31, 2.92)	1.08(0.00,2.62e+28)
0.32 (0.00, 7.68e+27)	0.32 (0.13, 1.83)	1.41 (0.00, 3.38e+28)	CT+CFT+ACU	3.02 (0.28, 32.55)	2.24 (0.38, 13.31)	1.44 (0.41, 5.06)	0.55 (0.12, 2.53)	1.33 (0.00, 3.20e+28)	1.34 (0.00, 3.20e+28)	1.53 (0.23, 9.95)
0.11 (0.00, 2.62e+27)	0.11 (0.01, 1.95)	0.47 (0.00, 1.15e+28)	0.33(0.03,3.57)	CT+CFT+EA	0.74 (0.05, 10.55)	0.48 (0.05, 4.92)	0.18 (0.02, 2.20)	0.44 (0.00, 1.09e+28)	0.44 (0.00, 1.09e+28)	0.51 (0.03, 7.66)

0.14 (0.00, 3.47e +27)	0.14 (0.03, 1.65)	0.63 (0.00, 1.52e +28)	0.45(0 .08,2. 65)	1.35 (0.09, 19.16)	CT+C FT+A P	0.64 (0.11, 3.60)	0.25 (0.04, 1.69)	0.59 (0.00, 1.45e +28)	0.60 (0.00, 1.45e +28)	0.68 (0.07, 6.24)
0.22 (0.00, 5.33e +27)	0.22 (0.10, 1.51)	0.98 (0.00, 2.34e +28)	0.69(0 .20,2. 44)	2.10 (0.20, 21.63)	1.56 (0.28, 8.71)	CT+C FT+H PN	0.38 (0.09, 1.64)	0.92 (0.00, 2.22e +28)	0.93 (0.00, 2.22e +28)	1.06 (0.17, 6.53)
0.58 (0.00, 1.39e +28)	0.58 (0.18, 1.91)	2.55 (0.00, 6.12e +28)	1.8(0. 40,8.2 3)	5.44 (0.45, 65.32)	4.04 (0.59, 27.60)	2.60 (0.61, 11.04)	CT+C FT+ MOX	2.40 (0.00, 5.80e +28)	2.42 (0.00, 5.80e +28)	2.76 (0.37, 20.51)
0.24 (0.06, 1.96)	0.24 (0.00, 5.82e +27)	1.06 (0.21, 5.39)	0.75 (0.00, 1.81e +28)	2.27 (0.00, 5.64e +28)	1.68 (0.00, 4.10e +28)	1.08 (0.00, 2.61e +28)	0.42 (0.00, 1.01e +28)	CT+H PN+B LT	1.01 (0.21, 4.79)	1.15(0 .00,2. 81e+2 8)
0.24 (0.12, 1.50)	0.24 (0.00, 5.70e +27)	1.05 (0.34, 3.23)	0.74(0 .00,1. 78e+2 8)	2.25 (0.00, 5.52e +28)	1.67 (0.00, 4.02e +28)	1.07 (0.00, 2.55e +28)	0.41 (0.00, 9.88e +27)	0.99 (0.21, 4.69)	CT+A CU	1.14(0 .00,2. 75e+2 8)
0.21 (0.00, 5.09e +27)	0.21 (0.04, 1.07)	0.93 (0.00, 2.24e +28)	0.65(0 .10,4. 26)	1.97 (0.13, 29.87)	1.47 (0.16, 13.40)	0.94 (0.15, 5.79)	0.36 (0.05, 2.70)	0.87 (0.00, 2.12e +28)	0.88 (0.00, 2.12e +28)	CT+C FT+N WM
